# Assessment of maternal anemia in rural Western China between 2001 and 2005: a two-level logistic regression approach

**DOI:** 10.1186/1471-2458-13-366

**Published:** 2013-04-19

**Authors:** Leilei Pei, Lin Ren, Duolao Wang, Hong Yan

**Affiliations:** 1 Department of Epidemiology and Health Statistics, School of Public Health, Xi’an Jiaotong University College of Medicine, No.76 West Yanta Road, P.O. Box 46, Xi’an, Shaanxi, 710061, P. R. China; 2 Department of Epidemiology and Population Health, London School of Hygiene and Tropical Medicine, London, WC1E 7HT, UK

## Abstract

**Background:**

There are multiple adverse effects of anemia on human function, particularly on women. However, few researches are conducted on women anemia in rural Western China. This study mainly aims to investigate the levels and associated factors of maternal anemia between 2001 and 2005 in this region.

**Methods:**

6172 and 5372 mothers with children under three years old were selected from 8 provinces in 2001 and from 9 provinces in 2005 respectively in Western China by means of a multi-stage probability proportion to size sampling method (PPS). The blood samples were tested and related socio-demographic information was obtained through questionnaires. A two-level logistic regression model was employed to identify the determinants and provincial variations of women anemia in 2001 and 2005.

**Results:**

The results indicated that the crude prevalence of women anemia in 2005 was higher than the rate in 2001(45.7% vs 33.6%). Based on the nationwide census data in 2000, the age-standardized prevalence of women anemia in the study were obtained as 38.0% in 2001 and 50.0% in 2005 respectively. Two-level logistic model analysis showed that compared to the average, women were more likely to be anemic in Guangxi and Qinghai in 2001 as well as in Chongqing and Qinghai in 2005; that women from Minority groups had higher odds of anemia in contrast with Han; that women with higher parity, longer breastfeeding duration and higher socioeconomic level had a lower rate of anemia, while age of women was positively associated with anemia. The positive correlation between women anemia and altitude was also observed.

**Conclusions:**

The study demonstrated that the burden of maternal anemia in rural Western China increased considerably between 2001 and 2005. The Chinese government should conduct integrated interventions on anemia of mothers in this region.

## Background

Anemia remains one of the most widespread health problems all over the world and affects about 1.62 billion people, which was characterized by reductions in hemoglobin concentration (HBC), red blood cell count (RBC), packed-cell volume (PCV), and subsequent impairment in meeting the oxygen demands of tissues [1,2]. Although anemia results from a wide variety of causes including folate or vitamin B-12 deficiency, infection, inflammation, heredity and so on, it is generally perceived that the most important contributing factor of anemia is iron deficiency, which accounts for about half of all anemia cases [3-5]. Because of depleting their iron stores by the end of their pregnancy, losing a large amount of blood during childbirth, secreting a large amount of nutrients in their breast milk, and recurrent menstrual loss, women of reproductive age are most at risk of anemia [6,7]. Many studies also showed that anemia could lead to not only impairment in work capacity and learning ability but also increases of morbidity from infections [8-11]. Especially severe anemia is closely related to a greater risk of death.

WHO had established an epidemiological criterion to classify areas with respect to the level of public health significant of anemia: low (prevalence of <15%), medium (15-40%) and high (>40%) in the world [12]. According to WHO global database on anemia for 1993–2005, approximately 468 million non-pregnant women were estimated to be anemic, with Africa having the highest prevalence of anemia, and Asia bearing the greatest absolute burden [1,13]. In China, anemia affected almost a third of the country’s lactating women mainly due to iron deficiency [14].

Western China has a total land area of 5.38 million square kilometers and a population of 287 million across following expression:12 provinces, where economic condition and health services are poor. In addition, Western China is significantly ethnically diverse with 44 ethnicities, which occupies nearly 80% of minorities in China. The degree of integration of ethnic minorities with each other and with Han ethnicity varies significantly from group to group. Nowadays, it is estimated that approximately 40 million people in Western China are still living in poverty, most of who are from minorities [15]. However, the information on women anemia is scarce in rural areas of Western China. Data on women anemia is important to guide appropriate targeted packages of interventions to address different types of women malnutrition. Therefore, Chinese Ministry of Health (MOH) and the United Nations Children’s Fund (UNICEF) conducted “Safe Motherhood” project in rural Western China from 2001 to 2005 [16]. On the basis of the survey data, the objectives of this study were to compare the prevalence of anemia among mothers with a child aged <3 years between 2001 and 2005 and identified the risk factors for mothers anemia.

## Methods

### Study design

Two population-based cross-sectional studies were conducted from June to August in 2001 and 2005 respectively in rural Western China. 41 counties of 8 provinces in 2001 and 40 counties of 9 provinces in 2005 were all determined by the Chinese Ministry of Health (MOH) and the United Nations Children’s Fund (UNICEF) rather than sampled randomly. Of them, there were the same 8 provinces and 29 counties in both 2001 and 2005. After a multi-stage probability proportion to size sampling (PPS) method was adopted, 5 townships in the chosen counties and then four villages from each township were selected randomly. As an example, how to select five townships from a chosen county is presented as follows. Firstly, we sort all townships in a county from smallest to largest according to the population size, and then calculate the accumulated population size. Suppose that there is a total population of 202000 in a township, and the sample interval can be calculated as 202000/5 = 40400. After that, a random number is determined based on the banknote serial number of Renminbi (Chinese currency). The random number consists of the last digits of this serial number, which has the same length as that of the sampling interval. For example, we have selected the serial number 98272809 from Renminbi banknote, and then the serial number 72809 is determined. Next, the first selection number is 32049 by taking the absolute value of difference between the random number and sample interval. If the first selection number is contained in the accumulated population size of this township, the first township is included in this study. The second selection number is the first selection number plus the sampling interval, and so the second township is selected. The third, fourth and the fifth townships are chosen with the same method. Similarly, four villages are selected from each chosen township. The sampling interval for village selection is calculated by dividing the total population size of a township by four. Finally, eight mothers with children under three years old were selected randomly from each chosen village. The main inclusion criteria were that mothers (>15 years old) were non-pregnant with a strong willingness of participate and without other diseases during the survey.

After this study was fully explained to participants, the informed consent was obtained from them. Socioeconomic and demographic characteristics were collected from the questionnaires. The protocol was also reviewed and approved by the Human Research Ethics Committee of the Xi’an Jiaotong University College of Medicine.

### Hemoglobin measurement

Capillary blood was collected from each participant using a finger-prick method to extract three drops of blood from the left middle and/or ring finger. After the first two drops of blood were wiped away, the third drop of blood was used immediately for testing hemoglobin concentration (HBC) by Hb photometer (B-Hemoglobin, precision of 0.1 grams/decilitre, Hemocue AB, Sweden). The anemia of nonpregnant women in this study was defined as HBC less than 12.0 g/dl. Besides, three levels of severity of anemia were also distinguished: mild anemia (10.0-11.9 g/dl), moderate anemia (7.0-9.9 g/dl), and severe anemia (less than 7.0 g/dl) [17]. Many studies had shown that HBC increased rapidly with altitude especially at altitude of higher than 1000 m sea level [18-20]. Previous study had also found that Centers for Disease Control (CDC) method might be more suitable to adjust altitude in Western China compared with other methods [21]. Thus, in this study CDC method [18] was solely utilized with the following expression:

$$\begin{array}{cc} \Delta \mathrm{Hb}= & -0.032\times \left(\mathrm{Alt}\times 0.0033\right)+0.022\\ & \times {\left(\mathrm{Alt}\times 0.0033\right)}^{2} \end{array}$$

Where Δ*Hb* was the increment of HBC by increased altitude above sea level, and *Alt* was the altitude (m).

### Quality control

A pilot study was carried out to pretest all questionnaires and procedures and then the detailed interviewer guides were developed. The interviewers were trained to standardize questionnaires administration and HBC measurements at least one week before commencement of the survey. After signing the informed consent form, all participants were interviewed face-to-face by means of the pre-coded structured questionnaires to collect the information on socio-demographic characteristics of mothers. Meanwhile, blood sample collections of mothers were carried out to measure their HBC.

Nineteen investigation teams from Xi’an Jiaotong University College of Medicine were established for these counties. Each team consisted of four or five members and one supervisor. During the survey, all fieldworkers were closely monitored by their supervisors and randomly examined. Participants were re-interviewed immediately when errors and/or missing values were detected.

### Statistical analysis

#### Explanatory variables

Mothers’ anemia was considered as a unique outcome variable in the study. We considered the certain individual characteristics of mothers as the proximate covariates of anemia. Individual-level covariates included ethnicity, which consisted of Han, Tibet, Uighur, Hui, Zhuang and Others; parity (1, 2, or >2); mothers’ age; and breastfeeding duration. The county-level covariate was altitude of county (<500 m, 500 ~ 1500 m, or >1500 m). Due to lack of income data of each household, wealth index was constructed through the principal component analysis for assessing economic status of the household [22]. The principal component analysis synthesized information on a set of household assets and living conditions: the ownership of a car, television, bicycle and motorcycle; the availability of clean water; the resources of household income and so on. Based on the tertiles of the first principal component, the socioeconomic status of the households was classified into 3 levels indicating the poorest, middle and wealthiest households.

#### Two-level logistic regression

Considering the hierarchical structure of the study sample, a two-level logistic model was applied to assess the influences of the covariates on the outcome variable. The observed responses *y*_*ij*_ are proportions with the standard assumption that they are binomially distributed *y*_*ij*__*=*_*Bin*(*π*_*ij*_, *n*_*ij*_), where *n*_*ij*_ is the denominator for the proportion. The variance-components model correct for the problem of correlated observations in a cluster, by introducing a random effect at each cluster. Consider the 2-level logistic variance model where the expected proportion is modeled using a logit link function.

(1) $$P_{\mathrm{ij}}\;=\;{\left\{1\;+\;\exp\left(-\left[\beta_{0j}\;+\;\beta_{1}X_{1\mathrm{ij}}\;+\cdots +\beta_{m}X_{\mathrm{mij}}\right]\right)\right\}}^{-1}$$

Where, $\beta_{0j}=\beta_{0}+u_{0j},\;u_{0j}\sim \left(0,\sigma_{u_{0}}^{2}\right),\;\mathrm{var}\left(P_{\mathrm{ij}}\right)=\delta \pi_{\mathrm{ij}}\left(1-\pi_{\mathrm{ij}}\right)/n_{\mathrm{ij}},\;\delta$ was scale parameter. The model could also be expressed as follows,

(2) $$\log\mathrm{it}\left(P_{\mathrm{ij}}\right)=\beta_{0}+\beta_{1}X_{1\mathrm{ij}}+\cdots +\beta_{m}X_{\mathrm{mij}}+u_{0j}$$

In the study, level 1 represented the individual and level 2 was the county. *P*_*ij*_ was the proportion of mothers anemia from the j^th^ county in the i^th^ individual. *β*_1⋯ _*β*_m_ were the regression coefficients corresponding to the effects of fixed covariates, *X*_1*ij*_⋯ *X*_*mij*_ were the observed characteristics of the mothers and the county. The random effects were presented as random variance with standard error (SE), and fixed effects as an odds ratio (OR) with 95% confidence interval (CI). The Variance Partition Coefficient (VPC, %) was calculated as the proportion of variation that is attributable to the county-level sources of variation.

The data was entered into Epi Info Version 6.0 (CDC, Atlanta, GA, USA) by double entry. All analysis was performed using STATA Version 12.0 (Stata Corporation, College Station, TX, USA). Independent *t*-test was used to compare the means of two different populations, and the comparison between two independent proportions was made by means of *χ*^2^ test. In addition, effect coding was used in the two-level logistic analysis to estimate the differences among 10 provinces with the grand mean of the 10 provinces as the reference category.

## Results

### Baseline characteristics

In the survey population, 388 mothers in 2001 and 1028 in 2005 were not interviewed. The reasons were that they were busy with farming when surveyed, or refused to have their HBC checked due to fear of pain or religious beliefs. Ultimately, a total of 6172 eligible participants in 2001 and 5372 in 2005 were obtained in the survey.

Of the participants, more than half of mothers were of Han ethnicity in 2001 and 2005 (65.4% vs 57.4%, P < 0.001). The mean age were 26.8 years old (range 15–50 years) in 2001 and 27.3 years (range 16–50 years) in 2005, in which there was a statistically significant difference between the two years (*P* < 0.001). The average breastfeeding duration of mothers across the whole population generally indicated a shift toward higher levels in 2005 compared to 2001(15.1 vs 17.0 months, P < 0.001). The altitude of the surveyed areas ranged from 52 to 3800 meters with a mean of 1216 meters above sea level.

### Prevalence of anemia between 2001 and 2005

Table 1 showed the prevalence of mothers’ anemia of different degree among socioeconomic characteristics and among different provinces between 2001 and 2005. The results indicated that after adjustment of altitude by CDC method, the average HBC of mothers were decreased from 12.5 ± 1.5 g/dl in 2001 to 12.0 ± 1.6 g/dl in 2005 (not shown in Table 1). The overall crude prevalence of mothers anemia was 33.6% in 2001, while the rate reached as high as 45.7% in 2005 (*P* < 0.001) with a large growth of about 12.1%. Among the participants, the prevalence of mild anemia and moderate anemia were increased by 6.6% and 5.6% respectively in 2005. However, it was not found that the rate of severe anemia significantly rose in this period. Based on the nationwide census data in 2000, the age-standardized prevalence of women anemia in the study were obtained as 38.0% in 2001 and 50.0% in 2005 respectively.

**Table 1:** **Prevalence of anemia of mothers with children under-three years by years among baseline characteristics **^**a**^

***Characteristics***^***b c***^	***2001***					***2005***				
	***Sample***	***Any anemia (<12.0 g/dl)***	***Mild anemia*** ***(10.0-11.9 g/dl)***	***Moderate anemia (7.0-9.9 g/dl)***	***Severe anemia (<7.0 g/dl)***	***Sample***	***Any anemia (<12.0 g/dl)***	***Mild anemia (10.0-11.9 g/dl)***	***Moderate anemia (7.0-9.9 g/dl)***	***Severe anemia (<7.0 g/dl)***
		***No.(%)***	***No.(%)***	***No.(%)***	***No.(%)***		***No.(%)***	***No.(%)***	***No.(%)***	***No.(%)***
**Region**										
Gansu	635	163(25.7)	137(21.6)	25(3.9)	1(0.2)	256	50(19.5)	44(17.2)	4(1.6)	2(0.8)
Guangxi	823	374(45.4)	318(38.6)	51(6.2)	5(0.6)	734	383(52.2)**	330(45.0)*	52(7.1)	1(0.1)***
Guizhou	-	-	-	-	-	455	252(55.4)	224(49.2)	28(6.2)	0(0.0)
Inner Mongolia	816	227(27.8)	209(25.6)	17(2.1)	1(0.1)	590	177(30.0)	161(27.3)	15(2.5)	1(0.2)
Ningxia	832	202(24.3)	182(21.9)	18(2.2)	2(0.2)	550	187(34.0)***	165(30.0)***	22(4.0)*	0(0.0)
Qinghai	957	467(48.8)	350(36.6)	108(11.3)	9(0.9)	615	375(61.0)***	198(32.2)	175(28.5)***	2(0.3)
Sichuan	688	221(32.1)	195(28.3)	24(3.5)	2(0.3)	640	268(41.9)***	236(36.9)**	29(4.5)	3(0.5)
Xinjiang	666	210(31.5)	179(26.9)	29(4.4)	2(0.3)	1028	467(45.4)***	351(34.1)***	111(10.8)***	5(0.5)
Chongqing	755	212(28.1)	192(25.4)	20(2.6)	0(0.0)	504	295(58.5)***	177(35.1)***	118(23.4)***	0(0.0)
**Ethnicity**										
Han	4036	1208(29.9)	1062(26.3)	140(3.5)	6(0.1)	3078	1303(42.3)***	1029(33.4)***	270(8.8)***	4(0.1)
Tibet	446	189(42.4)	142(31.8)	42(9.4)	5(1.1)	317	143(45.1)	90(28.4)	51(16.1)**	2(0.6)
Uighur	569	190(33.4)	161(28.3)	27(4.7)	2(0.4)	621	246(39.6)*	197(31.7)	46(7.4)	3(0.5)
Hui	334	128(38.3)	101(30.2)	26(7.8)	1(0.3)	260	106(40.8)	59(22.7)*	45(17.3)***	2(0.8)
Zhuang	398	199(50.0)	159(39.9)	36(9.0)	4(1.0)	334	196(58.7)*	155(46.4)	40(12.0)	1(0.3)
Others	386	162(42.0)	137(35.5)	21(5.4)	4(1.0)	751	453(60.3)***	351(46.7)***	100(13.3)***	2(0.3)
**Parity**										
1	3718	1272(34.2)	1076(28.9)	183(4.9)	13(0.3)	3127	1424(45.5)***	1094(35.0)***	326(10.4)***	4(0.1)
2	2016	673(33.4)	579(28.7)	88(4.4)	6(0.3)	1921	886(46.1)***	690(35.9)***	190(9.9)***	6(0.3)
>2	436	131(30.0)	107(24.5)	21(4.8)	3(0.7)	324	144(44.4)***	102(31.5)*	38(11.7)**	4(1.2)
**Altitude, m**										
<500	1521	477(31.4)	409(26.9)	65(4.3)	3(0.2)	1222	545(44.6)***	445(36.4)***	98(8.0)***	2(0.2)
500 ~ 1500	2370	651(27.5)	574(24.2)	69(2.9)	8(0.3)	2430	1098(45.2)***	832(34.2)***	262(10.8)***	4(0.2)
>1500	2281	948(41.6)	779(34.2)	158(6.9)	11(0.5)	1720	811(47.2)***	609(35.4)	194(11.3)***	8(0.5)
**Wealth index**										
Poorest	1956	707(36.1)	594(30.4)	108(5.5)	5(0.3)	1656	759(45.8)***	581(35.1)**	173(10.4)***	5(0.3)
Middle	2370	773(32.6)	665(28.1)	98(4.1)	10(0.4)	2108	970(46.0)***	715(33.9)***	250(11.9)***	5(0.2)
Wealthiest	1846	596(32.3)	503(27.2)	86(4.7)	7(0.4)	1608	725(45.1)***	590(36.7)***	131(8.1)***	4(0.2)
**Total**	6172	2076(33.6)	1762(28.5)	292(4.7)	22(0.4)	5372	2454(45.7)***	1886(35.1)***	554(10.3)***	14(0.3)

^a^ Values are the number of anemic mothers and the prevalence rate of anemia is included in the bracket.

^b^ Missing values: 3 for ethnicity and 2 for parity in 2001; 11 for ethnicity in 2005.

^c^ Differences in women anemia between 2001 and 2005 were statistically analyzed by *χ*^2^ test. *denoted *P* < 0.05, ** *P* < 0.01, ***P < 0.001.

The prevalence of anemia in 2005 was higher than the rate in 2001 nearly within all subgroups and provinces. In all the provinces except Gansu and Guizhou, the proportions of women with anemia had increased considerably between 2001 and 2005, with the highest growth rate of 30.4% in Chongqing. During the period, the incidence of anemia ascended rapidly in most ethnicities. It was noted that among different ethnicities, the growth rate of 18.3% in other Minority group was the highest in 2005, with the growth rates of 11.2% and 7.9% respectively for mild and moderate anemia. Among the subgroups of different parity, the increase rate of anemia for mothers with more than 2 parities between 2001 and 2005 was highest. At the same time, it could be observed that in areas of different altitudes, the prevalence of anemia in 2005 was higher as compared to 2001. Furthermore, a statistically significant difference in women anemia between 2001 and 2005 by socioeconomic levels was also shown in the study, with higher rates in 2005 in comparison with 2001.

### Factors associated with anemia

Table 2 presented the results from the two-level logistic regression of women anemia as the outcome variable in 2001 and 2005. In terms of random effects, the VPC was decreased from 16.5% (in the null model) to 10.2% (in the full model) in 2001 and similarly from 20.4% to 16.2% in 2005, suggesting a cluster effect still existed after controlling for other associated factors in both two years. The results revealed that the prevalence of women anemia strikingly varied among the 10 provinces in the two years; that as compared to the average in 2001, the odds of women anemia were higher in Guangxi (OR:2.32, 95%CI: 1.54,3.50) and Qinghai (OR:1.46, 95%CI:1.01,2.11), and lower in Ningxia (OR:0.58, 95%CI: 0.40,0.83); that as compared to the average in 2005, the odds of women anemia were higher in Chongqing (OR:2.00, 95%CI: 1.03,3.85) and Qinghai (OR:1.86, 95%CI: 1.00,3.48), while the odds were lower in Gansu (OR:0.31, 95%CI: 0.13,0.74) and Inner Mongolia (OR:0.49, 95%CI: 0.26,0.93). For individual characteristics, women from Uighur ethnicity were more likely to be anemic compared to those from Han ethnicity in 2001, and women from Zhuang and others ethnicity had higher odds of anemia in comparison with Han in 2005. In these two years, women with higher parity were more likely to be anemic than those with one parity (OR: 0.76, 95%CI: 0.57, 0.99 in 2001 and OR: 0.84, 95%CI: 0.71, 0.98 in 2005). In 2001 and 2005, breastfeeding duration was inversely associated with women anemia (OR: 0.99, 95%CI: 0.98, 1.00; OR: 0.995, 95%CI: 0.991, 0.999), while age of women was positively correlated with anemia (OR: 1.02, 95%CI: 1.01, 1.04). The higher wealth index of households, the lower prevalence of women anemia in 2001 (OR: 0.90, 95%CI: 0.80, 0.99), but a similar positive correlation between them was not found in 2005. At the county level, the incidence rate of anemia increased with altitude, at which with >1500 m the odds of anemia was highest (OR: 2.47, 95%CI: 1.58, 3.84 in 2001 and OR: 1.86, 95%CI: 1.05, 3.64 in 2005).

**Table 2:** **Factors related to anemia: results from two-level logistic regression in 2001 and 2005 **^**a**^

**Characteristics**	**2001**		**2005**	
	**OR(95%CI)**	***P*-value**	**OR(95%CI)**	***P*-value**
**Null model**				
*u*_*0j*_	0.7 ± 0.1	<0.001	0.8 ± 0.1	<0.001
VPC	16.5%	-	20.4%	-
**Full model**				
**Fixed part**				
**Provinces**^**b**^				
Ningxia	0.58(0.40,0.83)	<0.01	0.63(0.33,1.22)	0.173
Xinjiang	0.63(0.34,1.14)	0.124	1.14(0.62,2.11)	0.663
Gansu	0.77(0.51,1.15)	0.201	0.31(0.13,0.74)	<0.01
Inner Mongolia	0.85(0.59,1.23)	0.386	0.49(0.26,0.93)	<0.05
Sichuan	1.04(0.72,1.51)	0.83	1.21(0.68,2.17)	0.515
Guizhou	-	-	1.56(0.82,2.96)	0.174
Chongqing	1.20(0.80,1.82)	0.378	2.00(1.03,3.85)	<0.05
Qinghai	1.46(1.01,2.11)	<0.05	1.86(1.00,3.48)	<0.05
Guangxi	2.32(1.54,3.50)	<0.001	1.31(0.71,2.44)	0.387
***Individual characteristics***				
**Ethnicity**				
Han	1.00		1.00	
Tibet	1.29(0.92,1.79)	0.136	1.23(0.79,1.92)	0.355
Uighur	2.35(1.28,4.30)	<0.01	1.42(0.74,2.73)	0.288
Hui	1.36(0.99,1.87)	0.060	0.57(0.40,0.82)	<0.01
Zhuang	1.06(0.68,1.63)	0.806	2.09(1.34,3.26)	<0.01
Others	1.26(0.93,1.70)	0.133	1.42(1.05,1.93)	<0.05
**Parity**				
1	1.00		1.00	
2	0.91(0.79,1.05)	0.194	0.84(0.71,0.98)	<0.05
>2	0.76(0.57,0.99)	<0.05	0.83(0.62,1.12)	0.228
**Wealth index**				
Poorest	1.00		1.00	
Middle	0.93(0.81,1.06)	0.280	1.05(0.91,1.22)	0.496
Wealthiest	0.90(0.80,0.99)	<0.05	1.09(0.92,1.28)	0.313
**Mothers’ age, years**	1.00(0.99,1.03)	0.252	1.02(1.01,1.04)	<0.01
**Breastfeeding duration**	0.99(0.98,1.00)	<0.05	0.995(0.991,0.999)	<0.05
***County characteristics***				
**Altitude, m**				
<500	1.00		1.00	
500 ~ 1500	1.03(0.70,1.53)	0.868	1.19(0.69,2.04)	0.530
>1500	2.47(1.58,3.84)	<0.001	1.86(1.05,3.64)	<0.05
**Random part**				
*u*_*0j*_	0.4 + 0.1	<0.001	−1.7 ± 0.3	<0.001
VPC	10.2%	-	16.2%	-

Abbreviation: *VPC* variance partition coefficient, *u*_*0j*_, random effect.

^a^ Values were given as mean ± SD or odds ratio (95%CI) unless otherwise stated.

^b^ Effect coding was used for provinces. The reference group is the average of all 10 provinces.

## Discussion

The article, to our knowledge, is the first to investigate the prevalence of maternal anemia in 2001 and 2005 in rural Western China. In addition, the study also provides the updated information on related factors and even regional differences of women anemia in the two years. Our results indicated that the prevalence of anemia among mothers with children less than three years old was higher in 2005 compared to 2001. Besides, there were statistically significant differences in the prevalence of anemia among the 10 provinces and among socioeconomic characteristics in both two years.

The study showed that the average HBC of mothers was reduced from 12.5 ± 1.5 g/dl in 2001 to 12.0 ± 1.6 g/dl in 2005, both of which were also far below a national average value of 13.0 g/dl in 2002 [14]. In the meantime, it was found that the crude prevalence of anemia among mothers had an increase rate of 12.1% from 2001 to 2005. After adjusted by age of women based on the nationwide census data in 2000, the age-standardized prevalence of anemia was also higher in 2005 than the rate in 2001 (38.0% vs 50.0%). According to surveillance data available for 1995–2000 in the world, it was estimated that the overall prevalence of anemia fell by 0.16% per year for non-pregnant women [23]. However, among non-pregnant women during this period, trends in anemia worsened in sub-Saharan Africa (by 0.18% points per year), the Middle East and North Africa (by 0.06% points per year), and Southeast Asia (by 0.42% points per year) [23].

According to China Health and Nutrition Survey (CHNS) in 2002 [14], the whole prevalence of anemia in women of reproductive age was 19.9%. By contrast, it seems that the proportion was much higher in rural Western China in 2005(45.7%). The rate in rural Western China was similar to those in low-income and middle-income countries during the period. For example, more than forty-seven percent of mothers in Egypt were anemic in 2005 [24]; in India, the prevalence of anemia in lactating women was as high as 56.4% [25]; among non-pregnant women in Bangladesh, the rate of anemia had reached 46% in 2004 [26]. Unfortunately, the percentage of women with anemia in the study was higher in contrast with developed countries. For instance, studies showed that postpartum anemia occurred in 27% of women overall in the United States [26]; in Japan, the incidence of anemia among lactating women and non-pregnant/non-lactating women were 11.1% (HBC < 12 g/dl) and 15.7% respectively [27]. Research had confirmed that severe anemia was strongly associated with a higher risk of maternal mortality, with a 4.5-fold risk of maternal death in low-income countries [28,29]. In our study, however, the percentage of women with severe anemia was very low in both 2001 and 2005.

The two-level logistic regression analysis showed that women in higher socioeconomic level were less likely to have anemia in 2001. It was widely knowledged that poverty was relevant to limited education, insufficient diet, poor housing conditions, and inadequate water and sanitation services, especially in developing countries [30]. The poor were more likely to be marginalized than their better-off peers in their ability to access health services because of constraints in financial and administrative resources. In addition, extreme poverty could greatly limit their ability to purchase a wide variety of food and nutrients, and increase the risk for infectious diseases and parasitic infestations. It is well documented that women from the wealthier households or with higher education had lower risk for anemic than those from poor households or with lower education [23].

In the study, the higher age of mothers, the greater the risk for anemia. However, the association of anemia with parity was just the opposite. The relationships between anemia and parity and age were also observed in some low-income countries [23,28]. Poor socioeconomic situation was known to be associated with a number of factors such as high parity and short birth interval. It was found that due to the long-lasting effects of age and parity on iron status, older women with higher parity might face an increased risk for anemia [23]. But in the study the adverse effect of higher parity on anemia had been not confirmed.

Breastfeeding duration was negatively correlated with women anemia. Previous study had shown that lactation was closely related to a delay in the normal return of menses [31]. Because iron losses in breast milk were only about half that lost during menstruation, the amenorrhea could decrease demand for iron. Another research also found that lactating women were more likely to take iron or iron-containing supplements post partum and to take them longer than nonlactating women [32]. Therefore, Breastfeeding for a longer duration appeared to have significant benefits for mothers’ health.

A difference in anemia among different ethnicities reflected the differential exposure to the risk factors of anemia. It was perceived that due to some historical reasons, Minority persons made up a disproportionally large majority of the poverty population in rural Western China. In China, because many minorities were not subject to birth restrictions as severe as those for Han majority [15], there was a larger household size among Minority households. Furthermore, the average education periods of Minority ethnic adults were shorter than Han ethnicity peers [15]. A synergy of these factors could contribute to the widespread ethnic differences.

The provincial differences of anemia were also clearly demonstrated in Western China. Among different provinces, not only socioeconomic status of households but also peoples’ knowledge, attitudes and beliefs of health, were closely linked to anemia. In the study, anemia tended to occur among low-income provinces, such as Guangxi and Qinghai in 2001 and Chongqing and Qinghai in 2005 [33]. In some provinces with backward economic and social basis, such as Ningxia in 2001 and Gansu in 2005, however, the rates of women with anemia were lower than the average value. Possible explanation was that most participants in the two provinces were from Han ethnicity (80.0% in Ningxia in 2001 and 99.6% in Gansu in 2005), and a mass of Muslims were included in Ningxia in 2001 (19.3%) that was determined as a protective factor for anemia in the last study [30]. On account of the cross-sectional design in the study, other reasons had not still been confirmed yet. Past work had also shown that higher altitude had significant effects on HBC [18-20]. In the surveyed areas, similarly, higher altitude was also an important contributing factor of mothers’ anemia. In rural Western China, the high-altitude region commonly has harsh natural environment, sparse population, poor health care services and underdeveloped economy. Therefore, these could help understand the higher rate of women with anemia at higher altitudes.

The strength of the current study is its large sample size. Although we have made large efforts in study design, sampling and statistical analysis, many limitations in the study may still exist. Firstly, because of the cross-sectional design in the two years, the causal relationships between socio-demographic characteristics and anemia were not confirmed, and thus interpretations of the conclusions should be made with caution. Secondly, the results were subject to many latent confounding factors. For example, occupation of mothers, diet and physical activity were unavailable in the study and may confound the findings.

## Conclusion

Maternal anemia continued to be an endemic problem of large magnitude in rural Western China, with a large increase between 2001 and 2005. The statistically significant differences in maternal anemia were found among the 10 provinces and among socio-demographic characteristics in 2001 and 2005. Our findings will have important policy implication for Chinese government to prevent maternal anemia in rural Western China.

## Competing interests

The authors declare that they have no competing interests.

## Authors’ contributions

All authors read and approved the final manuscript. L.P. and L.R. designed the prescription study, collected the data, conducted the data analysis and prepared the manuscript; H. Y. contributed to the design and analysis of the study and the preparation of the manuscript; and D. W. assisted with the data analysis and reviewed the manuscript.

## Pre-publication history

The pre-publication history for this paper can be accessed here:

http://www.biomedcentral.com/1471-2458/13/366/prepub
